# Supplementary material for: Improving Contraception Outreach through Human-Centered Design: A Pilot Study of the Ndingathe (“I Can”) Intervention in Rural Malawi
Source: PLoS One. 2025 Sep 24;20(9):e0332149. doi: 10.1371/journal.pone.0332149 (PMC12459769; doi:10.1371/journal.pone.0332149)
Supplement: Attachment — Submitted filename: Contraception Outreach in Rural Malawi.docx [file pone.0332149.s002.docx]

**Reviewer’s Comments:**

Journal: **PLOS ONE**

Manuscript title: **Feasibility, acceptability, and potential effectiveness of a human-centered design-derived intervention to improve community health workers’ contraception outreach in rural Malawi.**

First, thank you for the opportunity to review this manuscript. I will say this is a well-structured and timely study addressing a critical gap in contraceptive access in rural Malawi using a human-centered design approach. The mixed-methods design is appropriate. The triangulation of data is also commendable. However, the manuscript requires some improvements in some areas to meet the standard of a peer-reviewed publication. Below are my suggestions for improvement.

**Title and Abstract:**

- Page 1, Lines 5–6: The title is clear, but slightly long and can confuse. Remember, your work/data reads a pilot study. I will suggest you consider shortening it to read: *“Improving Contraception Outreach through Human-Centered Design: A Pilot Study in Rural Malawi.”*
- Page 2, Lines 30–54 (Abstract). The abstract is detail but exceeds typical word limits for many journals. It is above 300 words and its current state stresses readers. I will suggest you consider trimming descriptive content, especially in the “Methods” and “Results” subsections, without losing key details. Make it smart and interesting to readers.

**Introduction:**

- Page 4, Lines 58–87: The introduction somewhat effectively outlines the problem but needs a clearer articulation of the *knowledge gap*. The authors should more explicitly state *why* previous approaches have not succeeded and how *Ndingathe* uniquely addresses this. You may wish to address the question of what’s novel beyond combining peer support and logistics support. Also, what ha previous studies focused on and what gap in the body of knowledge is this study bridging or filling.
- Additionally, please ensure that in-text references follow the PLOS ONE style, with reference numbers inserted *before* the full stop. This also apply to other sections of the manuscript. I recommend you consulting the PLOS ONE journal's author submission guidelines for detailed instructions on formatting and referencing

**Theoretical Framework:**

- Page 7, Lines 135–13: The “Contraceptive Agency Framework” is referenced, but there is no adequate explanation of what the framework entails. I will suggest you provide a concise summary of the framework or a figure caption for “Figure 1” that explains the mechanisms and constructs. This will help readers to understand better and be carried along.

**Study Design and Methodology:**

- Page 9, between lines 153–159: The use of a “reduced intervention package” as a quasi-comparator group is mentioned but not well developed. It doesn’t carry the readers along clearly as expected. Please expand the rationale and design of this comparison arm. If I may ask, were outcomes stratified by intervention exposure level (with vs. without EUs)? If so, please present those comparisons.
- Page 10, Around Lines 168–173: The sampling strategy is not fully described. How were the 450 clients selected? Was there any randomization or consecutive sampling? Please clarify recruitment criteria to assess risk of selection bias. This is very important, please.
- Page 12, Lines 230–236: The observation methodology you used slightly lacks detail. How were the 20 outreach sessions selected? Were observers standardized or trained for consistency? What made up the observation template? Was it only the number of clients that visited by time of day? Please address these in the methodology section.

**Data Analysis:**

- Page 13, Lines 237–244: On your analysis, while the statistical tests (Wilcoxon, t-tests) seem appropriate and I don’t have problem with you using them, but the manuscript does not address potential confounders or clustering effects (e.g., HSAs nested within districts). Could you please address this? Please address the limitation of not controlling for clustering or interdependence in responses. If clustering wasn't accounted for, acknowledge this as a limitation.
- Please indicate also how missing data were handled (e.g., non-responses, incomplete forms) in your study/analysis.

**Results:**

- The use of chart in the presentation of your paired t-test may seem pictorial but lacking some key ingredients which are very important, and has pushed me to interrogate the presentation and completeness your statistical test results, especially given the reliance on paired t-tests and Wilcoxon signed-rank tests to establish pre-post differences.
- I notice inadequate presentation of paired t-test and Wilcoxon results across the results. For example, on Page 20, Figure 3 (HSAs’ role conflict, overload, job satisfaction), Page 21, Figure 5 (Client-reported fear before/after interacting with experienced users) and other Figures where you used paired t-tests and Wilcoxon signed-rank tests. The manuscript only presents mean values and p-values via bar charts. While this is helpful for visual readers, bar charts alone are not sufficient to fully interpret statistical results. In fact, key statistical reporting elements are missing, including:
  - Mean difference (or median difference for Wilcoxon)
  - Standard deviation or standard error
  - 95% Confidence Interval (CI) around the difference
  - Test statistics (e.g., t-value, z-score, or W-statistic for Wilcoxon)
  - Sample size (n) per comparison group clearly labelled

Without this information, readers cannot assess the precision, effect size, or practical significance of the findings, which you will agree with me if you carefully check through your results presentations. As you are aware p-values alone which you used tell us whether a difference is statistically significant but not the magnitude or relevance of that difference.

- I will suggest you use Tables instead of charts format. The Table format allows quick comparison, gives effect size estimates, includes sample size, CI, and test details. In fact, it complies with EQUATOR Network (e.g., CONSORT, STROBE) reporting standards.
- If at all visual presentation is retained, annotate bar charts with exact mean/median values, n, and 95% CI bars is preferred. You should include footnotes stating the test used and p-values. Avoid using bar graphs for ordinal scales (e.g., Likert-based fear levels). Boxplots or stacked bar charts showing distribution would be more appropriate for Wilcoxon tests. Despite this, I will still suggest, you use table format to align with scientific writing. It is not a policy brief. It is an original research manuscript.
- You interpretation also need to improve. Add interpretation of Practical Significance. May it not only statistically significant, but meaningful? The authors should discuss what the results mean in practice to be clinically or programmatically relevant?
- Page 15, Line 284: Statement: “...suggesting that offering afternoon clinics was helpful...” This is an inference not strongly supported by comparative data. I would have said authors should consider including pre-intervention time-use data or more rigorous client preference analysis. However, I do hope, a table presentation of the paired test results showing all the statistical test information should address this.
- Page 16, Lines 295–303: The quote from the 22-year-old client is strong. However, please consider providing more thematic synthesis across quotes to highlight commonalities, not just illustrative anecdotes. Using only a quote from a respondent to address this very theme is disturbing. Since the authors used a software for analysis, I was expecting to see thematic analysis running through quotes. Could you address this, please?
- Page 20, Line 369–371 (Fig. 3): The reductions in role conflict and overload are statistically significant, but the clinical or programmatic relevance of these changes is not discussed. In addressing this, please provide effect sizes or interpretation of whether these are considered “meaningful” changes in workload and motivation. I want to believe your table presentation of results will assist in this aspect also.

**Discussion:**

- Page 26, Lines 499–507: The discussion reiterates findings but still lacks important reflection on what did *not* work. For example, the issue of delayed lunch allowances (page 18, lines 346–348) should be discussed more critically in terms of sustainability and implementation fidelity.
- On Page 27, Lines 525–529, the authors mention continued mnemonic use after the pilot, which is encouraging. However, it's unclear whether this was tracked systematically or anecdotal. Please clarify whether this finding was emergent from interviews or formally evaluated.
- Please also support your findings with previous studies showing aspects of agreements and disagreements with past studies. This is very important.

**Limitations:**

- Page 28, Lines 559–568: The limitations are appropriately acknowledged which are good, but the absence of a control group should be emphasized more strongly as a major constraint to attributing effects to the intervention. I know that you mentioned control group but clearly state that is a major constraint to attributing effects to the intervention.

**Writing and Clarity:**

Here, I will say that there are some aspects of the manuscript that contain grammatical errors or awkward phrasing, for example:

- Page 9, Line 108: “so that there would not be any overlap”. Consider rephrasing to read “to avoid overlap in outreach areas.”
- Page 27, Line 546: “...generally well-received”. Please also consider saying “broadly acceptable and culturally appropriate.”

I will suggest that a thorough copyedit is needed to correct minor grammar and enhance readability.

**Figures and Tables:**

- For your Figure 1, I will suggest you ensure the figure is well-labelled and visually communicates the causal logic between intervention components and contraceptive agency outcomes.
- For your Tables 1 and 2 (Page 14), though they are information, I will still suggest you consider including a footnote indicating how missing data were handled (e.g., non-responses, incomplete forms). I have also included this also be addressed in methodology.

**References:**

Again, please ensure that in-text references follow the PLOS ONE style, with reference numbers inserted *before* the full stop. Additionally, in the reference list, include the full DOI (https://doi.org/) or web address where applicable. I recommend consulting the journal's author submission guidelines for detailed instructions on formatting and referencing
