# Supplementary material for: Improving Contraception Outreach through Human-Centered Design: A Pilot Study of the Ndingathe (“I Can”) Intervention in Rural Malawi
Source: PLoS One. 2025 Sep 24;20(9):e0332149. doi: 10.1371/journal.pone.0332149 (PMC12459769; doi:10.1371/journal.pone.0332149)
Supplement: Attachment — Submitted filename: Response to Reviewers.docx [file pone.0332149.s004.docx]

**Response to reviewers*:*** Improving Contraception Outreach through Human-Centered Design: A Pilot Study of the Ndingathe (“I Can”) intervention in Rural Malawi

**Date of resubmit: July 10, 2025**

**Responses to Academic Editor Comments**

1. **Abstract**: Add a sentence or two about data analysis.

**Response**: We added a sentence about data analysis on page 2 line 40-41 of the abstract.

**New text**: “We analyzed quantitative data using descriptive and inferential statistics. We conducted a thematic analysis of qualitative data.”

2. **Introduction,** Page 4, Lines 67-69: The current text states, "These workers are often people’s first point of contact for health-related issues and are instrumental in promoting and providing contraceptive services, among many other primary care services." This should be reworded to clarify that Health Surveillance Assistants (HSAs) are the first point of contact for health-related issues in areas far from health centres (Primary health care delivery settings). This distinction is important because medical assistants are typically the first point of contact for health-related problems in rural areas. The primary role of HSAs is to promote health and prevent illnesses.

**Response**: Thank you for this important consideration. We reworded to clarify that HSAs are the first point of contact for people who living in rural areas on page 4 Line 70 of the introduction.

**New text in *italics***: “*In rural areas far from primary care clinics,* HSAs are often people’s first point of contact for health-related issues and are instrumental in providing contraceptive services.”

3. **Methods (Description of the intervention)-** correct the typo on page 7, line 136, where 'Ndingathe' has been written as 'Ndingathe'. Also, could you please provide more information about the contents of the observation template? Was the template structured or unstructured?

**Response**: We corrected the typo on page 7 line 141. We also added one sentence to page 11 lines 247-248 to provide more information about the contents of the structured observation template.

**New text**: “A structured observation template guided data collection of the number of clients arriving in the morning versus the afternoon.”

4. **Data Analysis**: Please provide some details on how the framework analysis was used to analyse the transcripts and develop the themes. Could you also explain how data from observation were analysed?

**Response:** Thank you for raising this important point. We provided details on how the framework analysis was used to analyze the transcripts and develop themes on page 12 lines 270-276.

**New text:** “After transcription, two researchers (JV and KG) independently reviewed the interview transcripts to familiarize themselves with the data. Relevant excerpts were extracted and organized into a matrix around the pilot intervention's key components: the work planning tool, bicycles and lunch allowances, the mnemonic “Sakufima,” and engagement with EUs. Within each element, excerpts were categorized according to feasibility and acceptability. Five researchers (IM, TJ, MN, JV, MK) were each assigned one component and used the matrix to develop themes. Organizing the data this way enabled systematic comparison across participant groups (e.g., clients, HSAs, stakeholders).”

**Response**: We also added a sentence to describe how the observation data was analyzed on page 11 lines 257-258.

**New text**: “For observational data, we calculated descriptive statistics of the number of women observed attending outreach clinics in the morning versus the afternoon.”

**Responses Reviewer #1 Comments**

Dear Authors,  
 
Thank you for submitting the data and analysis regarding a strategy to improve the availability and administration of injectable contraceptives in underserved rural regions lacking supplies and healthcare. The description of the strategy addresses acceptability, feasibility, and potential effectiveness through a training program led by experienced users and an accessibility strategy for self-administration of injectable contraceptives. Relevant effects were achieved according to the respondents and expected outcomes following the training and improvements in accessibility in situations where the HSA was frequently absent. Consideration of whether the effectiveness of contraception in a population was evaluated may be beyond the scope of the present evaluation design and should be avoided in the manuscript.

**Response:** Thank you for providing these detailed and thoughtful comments on our manuscript and allowing us the opportunity to clarify our study design. We have provided our responses below, including the text from our updated manuscript to highlight what we changed based on your feedback. We sought to study the preliminary effectiveness of the intervention, Ndingathe or ICAN, not the contraceptive method, DMPA-SC. We have clarified this in the following sections:

**New text:** In the introduction on page 5 lines 103-106 “By intentionally combining system-level supports for HSAs with peer support for women interested in self-injection, Ndingathe offers a novel, holistic approach to improving contraceptive access and strengthening women’s ability to choose self-injection if they prefer this method*.*”

**New text in *italics***: In the methods section on page 7 lines 149-154 “*The Ndingathe theory of action* (Figure 1) depicts the two main pathways by which we posit that the intervention will *improve* women’s contraceptive agency, *defined as agency related to making and acting on contraceptive decisions [25]* and increasing use of *self-injection* [22]. First, *optimizing the HSA workflow is intended* to improve service accessibility. Second, *strengthening self-injection support* is intended to increase clients’ self-efficacy and perceived control over contraceptive decision-making and self-efficacy to self-inject.”

5. Title: It is suggested to avoid the concept of effectiveness as there is no adequate population comparator nor an assessment of its contraceptive effect within the present report.

**Response:** We adjusted the title to avoid the concept of effectiveness on page 1 lines 5-6.

**New text:** The title was changed to *Improving Contraception Outreach through Human-Centered Design: A Pilot Study of the Ndingathe (“I Can”) intervention in Rural Malawi* per Reviewer #2’s recommendation.

6. **Abstract:** The comments regarding effectiveness also apply here.

**Response:** We clarified that we aimed to study the preliminary effectiveness of the Ndingathe intervention, so we retained language about this in the abstract on page 2, line 37. “To assess the feasibility, acceptability, and potential effectiveness of Ndingathe, we collected…”

**Methods:**

7. Regarding Consent, please address the considerations for Medroxyprogesterone Acetate concerning bone mineral density and potential long-term effects on the breast. If it was informed to patients.

**Response:** Clinical information about contraception was beyond the scope of our consent form, which focused on informing women of procedures and potential risks involved in taking part in a survey or interview with the research team. Our study focused on understanding women’s experiences with the Ndingathe intervention that all contraception clients (not just those who participated in our study) were receiving during the time period in which it was being piloted in the study districts.

8. It would be appropriate to describe how the temperature of medications was ensured during bicycle transport, as well as the separation from food items.

**Response:** Thank you for raising this point. Information on medication storage is beyond the scope of our intervention and pilot study, which did not alter HSAs’ transport of medications. Further, HSAs did not typically transport food; the allowance provided to them as part of the intervention was used by them to purchase food at a local restaurant near the outreach clinic. See text page 7 134-135 lines: “The lunch allowance given to HSAs as part of the intervention was intended to be used to purchase food at a local restaurant near the outreach clinic.”

9. The direct compensation provided to survey participants by the interviewers could be a variable influencing the acquired responses.

**Response:** We appreciate this concern. We included a sentence to account for courtesy bias from incentives given to survey participants in the Limitations section on page 27, line 588-590.

**New text**: “Lastly, we reimbursed clients, EUs, and stakeholders for their time answering interview questions, which may have influenced their response and introduced courtesy bias.”

10. The indirect compensation is linked to the decision to accept the injection. The reasons of individuals who did not accept the medication administration were not explored.

**Response**: Choice of injection was not an eligibility criterion for joining the study. Women who had interacted with HSAs or experienced users during an outreach clinic were eligible for surveys and interviews regardless of their contraceptive choice. We did not collect information on contraceptive choice as that was beyond the scope of our research questions.

11. The strategy is evaluated through self-reporting of injectable medication administration instructed by an experienced user but not validated by a healthcare professional at any point, which may have implications for the ultimate objective.

**Response:** We revised the sentence on page 7, line 141-143 to describe that healthcare professionals were also present during training.

**New text:** “In the pilot, the EU’s role complemented the training provided by HSAs, who ultimately retained authority over validating women’s correct SI DMPA-SC technique during training.”

12. Contraceptive effectiveness in a population cannot be measured with the proposed design, particularly without adequate monitoring of transport temperature and proper review of the administration technique by a healthcare professional.

**Response:** We did not study contraceptive effectiveness and do not report on it in this manuscript. We evaluated the potential promise of the Ndingathe intervention and share results on the potential effectiveness of the intervention to improve contraceptive access and strengthen women’s ability to choose self-injection if it is a method aligned with their preferences.

13. **Discussion:** A strategy that could be sustainable without continuous support is suggested. However, the implementation of the present strategy relies on HSA time (where it was not possible to work with all due to their other activities), the provision of a bicycle in good condition, the provision of lunch allowances, and verification of whether users persist even without compensation for their participation. The aforementioned suggests that the present strategy also requires ongoing investment and sustainability.

**Response:** Thank you for highlighting this important point. To better reflect the fact that scale-up of this intervention would require more information as to the resources required for sustainability, we have added a line in the Discussion: page 27 Lines 598-601

**New text in *italics****:* “While these initial results are encouraging, further testing is warranted to establish the solution's effectiveness in achieving its intended outcomes, such as improved contraceptive agency, *and to evaluate the minimum amount of resources required to ensure sustainability of the model*.”

**Overall Recommendation:**  
In general, the manuscript with the description of the strategy is suitable for publication with the suggested revisions.

**Responses to Reviewer #2’s Comments**

First, thank you for the opportunity to review this manuscript. I will say this is a well-structured and timely study addressing a critical gap in contraceptive access in rural Malawi using a human-centered design approach. The mixed-methods design is appropriate. The triangulation of data is also commendable. However, the manuscript requires some improvements in some areas to meet the standard of a peer-reviewed publication. Below are my suggestions for improvement.

**Response:** Thank you for your informative and thoughtful review and instructive comments that allowed us to strengthen our manuscript. We have provided our responses below, including the text from our updated manuscript to highlight what we changed based on your feedback.

**Title and Abstract:**

14. Page 1, Lines 5–6: The title is clear, but slightly long and can confuse. Remember, your work/data reads a pilot study. I will suggest you consider shortening it to read: *“Improving Contraception Outreach through Human-Centered Design: A Pilot Study in Rural Malawi.”*

**Response:** The title was changed to reflect a pilot study and include the name of the intervention.  The title was changed to *Improving Contraception Outreach through Human-Centered Design: A Pilot Study of the Ndingathe (“I Can”) intervention in Rural Malawi.*

15. Page 2, Lines 30–54 (Abstract). The abstract is detail but exceeds typical word limits for many journals. It is above 300 words and its current state stresses readers. I will suggest you consider trimming descriptive content, especially in the “Methods” and “Results” subsections, without losing key details. Make it smart and interesting to readers.

**Response**: We edited the abstract and tightened the language to make it more clear and engaging for readers.

**Introduction:**

16. Page 4, Lines 58–87: The introduction somewhat effectively outlines the problem but needs a clearer articulation of the *knowledge gap*. The authors should more explicitly state *why* previous approaches have not succeeded and how *Ndingathe* uniquely addresses this. You may wish to address the question of what’s novel beyond combining peer support and logistics support. Also, what have previous studies focused on and what gap in the body of knowledge is this study bridging or filling.

**Response:** We have revised the introduction to more clearly articulate the knowledge gap our study addresses. Specifically, we now include a review of prior interventions to highlight how most existing efforts have focused on either improving awareness raising of the product or provider training—often neglecting the systemic challenges HSAs encounter in conducting outreach and the psychosocial support women need to overcome well-documented fear of self-injection.

**New text**: “Despite the potential of DMPA-SC to ease contraceptive access barriers, the percentage of DMPA-SC users who self-inject in Malawi remains low (21% as of 2021), and qualitative research has identified fear of SI as a major concern among women [12,17]. Previous contraceptive counseling and message-based counseling interventions have shown promise for spreading awareness of the option to self-inject DMPA-SC, but the positive impact of these interventions on improving the quality of provider-patient interaction during contraceptive counseling is mixed [18,19]. Approaches to simultaneously address service delivery challenges faced by HSAs serving rural areas and the psychosocial needs of clients to follow through on their interest in self-injection is lacking. Other research has highlighted the need to not only ensure the availability of this contraceptive method option, but also to ensure women have support to overcome fears of injecting themselves and support for users to deal with potential side effect concerns, desire to switch/stop methods or other concerns [20,21].”

17. Additionally, please ensure that in-text references follow the PLOS ONE style, with reference numbers inserted *before* the full stop. This also apply to other sections of the manuscript. I recommend you consulting the PLOS ONE journal's author submission guidelines for detailed instructions on formatting and referencing

**Response:** We revised in-text references per the PLOS ONE journal’s author submission guidelines.

18. **Theoretical Framework:** Page 7, Lines 135–13: The “Contraceptive Agency Framework” is referenced, but there is no adequate explanation of what the framework entails. I will suggest you provide a concise summary of the framework or a figure caption for “Figure 1” that explains the mechanisms and constructs. This will help readers to understand better and be carried along.

**Response:** We have adjusted our explanation to clarify that the theoretical design of the intervention aims to optimize the HSA workflow to improve service accessibility and strengthen SI support to increase clients’ self-efficacy and perceived control over contraceptive decision-making and self-efficacy to self-inject. See page 7, lines 149-154.

**New text in *italics***: “*The Ndingathe theory of action* (Figure 1) depicts the two main pathways by which we posit that the intervention will *improve* women’s contraceptive agency, *defined as agency related to making and acting on contraceptive decisions [25]* and increasing use of *self-injection* [22]. First, *optimizing the HSA workflow is intended* to improve service accessibility. Second, *strengthening self-injection support* is intended to increase clients’ self-efficacy and perceived control over contraceptive decision-making and self-efficacy to self-inject.”

**Study Design and Methodology:**

19. Page 9, between lines 153–159: The use of a “reduced intervention package” as a quasi-comparator group is mentioned but not well developed. It doesn’t carry the readers along clearly as expected. Please expand the rationale and design of this comparison arm. If I may ask, were outcomes stratified by intervention exposure level (with vs. without EUs)? If so, please present those comparisons.

**Response**: We clarified in this section that due to resource limitations, we were only able to fund experienced users (EUs) for half of the HSAs. See page 8, lines 170-172. We do not, however, report on comparative findings in this paper.

**New text in *italics***: “The *other half of* HSAs were not paired with EUs *due to resource constraints,* and a reduced intervention package was delivered without the EU, but including the improved workflow and SI mnemonic.

20. Page 10, Around Lines 168–173: The sampling strategy is not fully described. How were the 450 clients selected? Was there any randomization or consecutive sampling? Please clarify recruitment criteria to assess risk of selection bias. This is very important, please.

**Response:** We described how 450 clients were selected using a convenience sample on page 9, lines 183-187.

**New text in italics**: “Research assistants attended outreach clinics *held by* Ndingathe-trained HSAs *to recruit* clients for *cross-sectional* surveys during the *pilot* period. Research assistants *selected a convenience sample of half of the trained HSAs in each district* and attended 1-2 of their outreach clinics *halfway through the intervention* to collect 10-20 client surveys per *selected* HSA. *Among the 30 HSAs whose outreach clinics we targeted, half had EUs present.”*

We also include additional information on the limitations in that section on page 27, lines 586-588

**New text**: “Sixth, we used convenience sampling for the client surveys, which may have introduced selection bias if participants did not represent all clients who attended clinics across the pilot period.”

21. Page 12, Lines 230–236: The observation methodology you used slightly lacks detail. How were the 20 outreach sessions selected? Were observers standardized or trained for consistency? What made up the observation template? Was it only the number of clients that visited by time of day? Please address these in the methodology section.

**Response:** We expanded the methodology section to provide greater detail on the outreach observation process on page 11 lines 247-248.

**New text**: “A structured observation template guided data collection of the number of clients arriving in the morning versus the afternoon.”

**Data Analysis:**

22. Page 13, Lines 237–244: On your analysis, while the statistical tests (Wilcoxon, t-tests) seem appropriate and I don’t have problem with you using them, but the manuscript does not address potential confounders or clustering effects (e.g., HSAs nested within districts). Could you please address this? Please address the limitation of not controlling for clustering or interdependence in responses. If clustering wasn't accounted for, acknowledge this as a limitation.

**Response:** Thank you for this suggestion. We have now explicitly acknowledged this limitation in that section of the manuscript on page 26, lines 572-576

**New text:** “This major constraint limits our ability to attribute observed changes directly to the intervention, as we cannot rule out confounding. Further, our quantitative analyses did not account for potential clustering of survey responses within a district (or, in the case of client surveys, by HSA). As such, our findings should be considered exploratory and would benefit from further validation using experimental or quasi-experimental methods.”

23. Please indicate also how missing data were handled (e.g., non-responses, incomplete forms) in your study/analysis.

**Response**: We have added a description of our approach to handling missing data in the Data Analysis section on page 12 lines 256-257: We also added footnotes to all tables specifying the amount of missing data for individual analyses.

**New text**: “Given relatively small amounts of missing data, we performed complete case analyses.”

**Results:**

24. The use of chart in the presentation of your paired t-test may seem pictorial but lacking some key ingredients which are very important and has pushed me to interrogate the presentation and completeness your statistical test results, especially given the reliance on paired t-tests and Wilcoxon signed-rank tests to establish pre-post differences.

I notice inadequate presentation of paired t-test and Wilcoxon results across the results. For example, on Page 20, Figure 3 (HSAs’ role conflict, overload, job satisfaction), Page 21, Figure 5 (Client-reported fear before/after interacting with experienced users) and other Figures where you used paired t-tests and Wilcoxon signed-rank tests. The manuscript only presents mean values and p-values via bar charts. While this is helpful for visual readers, bar charts alone are not sufficient to fully interpret statistical results. In fact, key statistical reporting elements are missing, including:

§  Mean difference (or median difference for Wilcoxon)

§  Standard deviation or standard error

§  95% Confidence Interval (CI) around the difference

§  Test statistics (e.g., t-value, z-score, or W-statistic for Wilcoxon)

§  Sample size (n) per comparison group clearly labelled

Without this information, readers cannot assess the precision, effect size, or practical significance of the findings, which you will agree with me if you carefully check through your results presentations. As you are aware p-values alone which you used tell us whether a difference is statistically significant but not the magnitude or relevance of that difference. I will suggest you use Tables instead of charts format. The Table format allows quick comparison, gives effect size estimates, includes sample size, CI, and test details. In fact, it complies with EQUATOR Network (e.g., CONSORT, STROBE) reporting standards.

**Response:** Thank you very much for this important point and suggestion. We have added new tables (Tables 3 and 4) to showcase quantitative findings as you suggested and removed the Figures we previously had that were less detailed (Figure 2-4). We also report on median difference from the Wilcoxon test (thank you for the suggestion) and specify sample sizes for each analysis in the tables.

25. If at all visual presentation is retained, annotate bar charts with exact mean/median values, n, and 95% CI bars is preferred. You should include footnotes stating the test used and p-values. Avoid using bar graphs for ordinal scales (e.g., Likert-based fear levels). Boxplots or stacked bar charts showing distribution would be more appropriate for Wilcoxon tests. Despite this, I will still suggest, you use table format to align with scientific writing. It is not a policy brief. It is an original research manuscript.

**Response**: We have removed the bar charts and converted mean/median values and confidence intervals into table format to better align with scientific reporting standards and your recommendation. These are shown in Tables 3-4, pages 15 and 19.

26. You interpretation also need to improve. Add interpretation of Practical Significance. May it not only statistically significant, but meaningful? The authors should discuss what the results mean in practice to be clinically or programmatically relevant?

**Response:** We discuss the programmatic relevance of our results in relation to contraception and self-injection in the discussion section, see page 25.

**New text (535-538)**: “We observed an increase from just under two-thirds to almost all HSAs reporting providing contraception outreach at least once per week—a large shift that could substantially bolster contraceptive access in the most remote areas if sustained.”

**New text (547-548):** “Ndingathe also showed promise for diffusing contraceptive SI technology, as evidenced by a substantially lower client-reported fear of injecting oneself after talking to an EU compared to before.”

27. Page 15, Line 284: Statement: “...suggesting that offering afternoon clinics was helpful...” This is an inference not strongly supported by comparative data. I would have said authors should consider including pre-intervention time-use data or more rigorous client preference analysis. However, I do hope, a table presentation of the paired test results showing all the statistical test information should address this.

**Response**: We did not have a more rigorous time-use data source pre-intervention. We have revised this sentence to instead focus on the implication of the observational finding, which is more accurately described as just confirming that the HSAs did indeed extend their clinics into the afternoon. See page 14, lines 306-309.

**New text in *italics****:* “Related to the first pathway in *the intervention’s* theory of action (Figure 1) on workflow components increasing service accessibility, *observational data during the pilot showed that HSAs stayed at outreach clinics into the afternoon, with 70% of women attending outreaches in the morning and 30% attending in the afternoon.* ~~suggesting that offering afternoon clinics was helpful in improving accessibility for some women~~.”

28. Page 16, Lines 295–303: The quote from the 22-year-old client is strong. However, please consider providing more thematic synthesis across quotes to highlight commonalities, not just illustrative anecdotes. Using only a quote from a respondent to address this very theme is disturbing. Since the authors used a software for analysis, I was expecting to see thematic analysis running through quotes. Could you address this, please?

**Response**: Thank you for this important and thoughtful comment. We have made several changes to strengthen the analytical depth of this section through new language to better connect quotes and theme and removal of extraneous quotes. We have included new text and original and/or new quotes below copied from our revised manuscript.

**New text in *italics;* quotes are original (page 15, lines 320-321) :** “*HSAs announced outreach clinic hours at village meetings and posted flyers to indicate their working hours in places where the outreach clinic is normally conducted.* In interviews, many clients described appreciating the extended outreach clinic hours because they could work or run errands in the morning and then access *contraceptive* services in the afternoon, providing greater convenience and flexibility in managing their daily responsibilities, as noted by *this* client”:

*They did well because, in the past, when you miss [the outreach clinic] in the morning and get there by 11, you could find them [HSAs] gone. But now, it is good because you can go to the farm, or you can go to the market, and still you can find the health surveillance assistants right there*. -22-year-old client, married, Ntchisi District

**New text; quotes are original (page 20, lines 416-432):** Another client shared how seeing the EU’s confidence in their own bodies helped shift her own perceptions:

*[The EUs] explained their experiences using Sayana. They didn’t face any issues with it; all showed they were fine in their bodies. Talking to them was encouraging and useful for us. – 31-year-old experienced user, married, Ntchisi District*

This peer-based reassurance was not only meaningful to clients but also recognized by HSAs as a powerful complement to provider-led instruction. Several HSAs reported that EUs were able to reassure women more effectively than they could on their own, and potentially also made their workload more efficient. One HSA from Ntchisi reflected:

*What made the work easier was that after we taught them, they were also meeting up the experienced users. Unlike when I was alone, it was taking time for a woman to self-inject. But when she sees the experienced user, they were motivated that this one is my fellow woman, then self-inject. – HSA, Ntchisi District*

By seeing women “like them” successfully self-injecting, clients felt more confident in their own abilities. This theme of peer reassurance and shared experience emerged frequently across interviews, underscoring the relational nature of learning and the unique value of integrating EUs into community-based self injection programs.

**New text; quotes are original (page 20-21, lines 436-452)**: “the mnemonic’s localization into the local language enhanced its clarity and relatability, fostering stronger comprehension among clients. One client articulated how the mnemonic’s components translated directly into practical steps, making the technique more approachable”:

*It is helpful because SA [from the mnemonic “Sakufima”] means mixing the medicine, press slowly, in that way it means we are pressing the injection until it finishes, so we saw that these words are good. – 37-year-old client, married, Mulanje District”*

This culturally adapted mnemonic thus served not just as a memory aid but also as a source of reassurance, translating technical steps into familiar language and concepts. This culturally adapted mnemonic thus served not just as a memory aid but also as a source of reassurance, translating technical steps into familiar language and concepts. Alongside the mnemonic, interaction with EUs emerged as a critical source of encouragement and confidence for clients, many of whom initially doubted their ability to self-inject correctly. The combination of step-by-step guidance and real-time reminders from EUs, often reinforcing mnemonic cues, were described as invaluable. Clients recognized that this peer support helped bridge knowledge gaps and mitigate anxieties:

*It was helpful that the experienced user was reminding us to shake [the injection device] vigorously. Then they said ‘handle it properly’ as we injected ourselves. In simple terms, they were reminding us of the process. To me, I think there was a benefit because without the experienced user we would not know. – 28-year-old client, married, Ntchisi district”*

**New text (page 22, lines 472-475)**: “Finally, the interpersonal aspect of EU follow-up fostered trusting connections, making clients feel comfortable reaching out for support and making the process of self-injecting seem less intimidating. Several clients contrasted the ease of communication with EUs against the more formal and sometimes cumbersome hospital environment.”

**New text (page 23, lines 489-491)**: “Related to the mnemonic, we identified two key themes. First, in terms of cultural resonance and memorability, the Sakufima mnemonic was easy for women to recall because it sounded like a person’s name or song, making it familiar and appealing.”

**New text (page 24, lines 508-510):** “Thematic analysis revealed that EUs played a powerful role in fostering both personal and community-level change. Many described feeling empowered as role models, taking pride in helping other women gain confidence in self-injection.”

**New text (page 24, 515-520)**: “Because EUs lived in the same villages as clients, their support was easily accessible and embedded in community life, making follow-up feel informal yet reliable. Finally, their strong desire to continue supporting others even after the pilot ended reflects a sense of community ownership and promise of sustainability. EUs, clients and HSAs also continued to use the mnemonic, Sakufima, beyond the intervention period, as reported spontaneously during follow-up interviews (not tracked systematically).”

**Removals**: We also removed three quotes that were duplicative of other quotes or information in the text to facilitate focus on the new synthesis information in the Results section.

29. Page 20, Line 369–371 (Fig. 3): The reductions in role conflict and overload are statistically significant, but the clinical or programmatic relevance of these changes is not discussed. In addressing this, please provide effect sizes or interpretation of whether these are considered “meaningful” changes in workload and motivation. I want to believe your table presentation of results will assist in this aspect also.

**Response**: Thank you for this important suggestion. We have added more to the Discussion section to reflect on the practical relevance of our findings.

**New text (page 25, lines 533-538)**: “The statistically significant decreases in HSAs’ role conflict and role overload after participating in the pilot represent meaningful changes important not only for HSAs’ quality of life but also signal potential to improve HSAs’ ability to offer quality healthcare to clients in rural areas. Indeed, we observed an increase from just under two-thirds to almost all HSAs reporting providing contraception outreach at least once per week—a large shift that could substantially bolster contraceptive access in the most remote areas if sustained.”

**Discussion:**

30. Page 26, Lines 499–507: The discussion reiterates findings but still lacks important reflection on what did *not* work. For example, the issue of delayed lunch allowances (page 18, lines 346–348) should be discussed more critically in terms of sustainability and implementation fidelity.

**Response:** This point is well taken. We added mention of the delayed lunch allowances and several additional findings from other researchers and studies, including systematic review of community health workers and incentive structures. Please see page 25 lines 544-546.

**New text:** “Our finding that the delayed lunch allowances affected HSAs’ morale demonstrates that paying attention to workers’ basic needs enables them to properly carry out their job duties [30].”

31. On Page 27, Lines 525–529, the authors mention continued mnemonic use after the pilot, which is encouraging. However, it's unclear whether this was tracked systematically or anecdotal. Please clarify whether this finding was emergent from interviews or formally evaluated.

**Response:** We have updated the manuscript to note that continued use of the mnemonic by HSAs after the pilot period was not formally tracked but rather emerged anecdotally during qualitative interviews with HSAs. This revision can be found on page 26 lines 557-559.

**New text (557-559):** “Further, continued dissemination of the mnemonic by HSAs after the pilot, underscores the mnemonic’s perceived value, ease of integration into routine practice, and suggests promise for long-term feasibility in SI contraception programs.”

32. Please also support your findings with previous studies showing aspects of agreements and disagreements with past studies. This is very important.

**Response:** This point is well taken. We have added more literature showing agreement and disagreement with our findings, including those detailed about lunch allowances and community health workers and peer support for contraceptive decision making in the discussion section and copied all the relevant sections below (page 24-26, lines 531-570)

**New text in italics:** Ndingathe is unique among *interventions* to improve contraceptive care *by* addressing fundamental structural issues that restrict women’s access to community health workers in the most rural areas [5]. *The statistically significant decreases in HSAs’ role conflict and role overload after participating in the pilot represent meaningful changes important not only for HSAs’ quality of life but also signal potential to improve HSAs’ ability to offer quality healthcare to clients in rural areas. Indeed, we observed an increase from just under two-thirds to almost all HSAs reporting providing contraception outreach at least once per week—a large shift that could substantially bolster contraceptive access in the most remote areas if sustained. Pilot findings suggest* Ndingathe helped mitigate transportation challenges by equipping HSAs with bicycles, which allowed them to reach remote areas *more frequently and stay for longer when also facilitated with lunch allowances. Bicycles* have shown promise for improving community health outreach in other rural settings in sub-Saharan Africa [28]. Furthermore, providing HSAs with lunch allowances *was appreciated by HSAs in the pilot,* and has been demonstrated in other studies to improve the efficiency of community health workers by addressing the financial *and physiological* barriers they face when conducting outreach [29]. *Our finding that the delayed lunch allowances affected HSAs’ morale demonstrates that paying attention to workers’ basic needs enables them to properly carry out their job duties [30]*.

*Ndingathe also showed promise for diffusing contraceptive SI technology, as evidenced by a substantially lower client-reported fear of injecting oneself after talking to an EU compared to before**. Hands-on support from peer experienced user of SI DMPA0-SC innovates on existing self-injection programs that focus primarily on spreading awareness of the option to self-inject and improving provider counseling [17,18]. A focus on peer support aligns with systematic reviews documenting that peer support may enhance the acceptability of new health behaviors by creating a trusting and relatable environment, especially for rural or other groups not well reached by the healthcare system [31,32]. In line with other research emphasizing the need for approaches to provide women* continued support and follow-up *for self-injection, particularly in settings where access to formal healthcare is limited [19], these pilot results suggest the* follow-up visits conducted by EUs *as part of Ndingathe may help women initiate or continue self-injection when they want to. Further, continued dissemination of the mnemonic by HSAs after the pilot, underscores the mnemonic’s perceived value, ease of integration into routine practice, and suggests* *promise for long-term feasibility in SI contraception programs.*

*The need to enhance contraceptive outreach clinics* provided by HSAs reflects broader trends in task-shifting in global health, where community health workers take on expanded responsibilities to alleviate workforce shortages *and reduce inequities in contraceptive access between rural and urban populations [8]. However, just as they are tasked to assist with the healthcare shortages in underserved areas, HSAs also face challenges that circumvent their ability to provide these much-needed services, including lack of transport while working in remote regions, limited supportive supervision, and frequent stockout of medical supplies [8–10].* In our study, the addition of EUs working alongside HSAs eased the burden on HSAs, allowing them to work more efficiently and *serve the needs of their clients, most of whom were from the same community as the EUs.* This peer-driven approach helped to alleviate the time pressures faced by HSAs, echoing findings by Ballard et al. that collaboration between community health workers and peer supporters *may* enhance the effectiveness of healthcare delivery [33].

33. **Limitations:** Page 28, Lines 559–568: The limitations are appropriately acknowledged which are good, but the absence of a control group should be emphasized more strongly as a major constraint to attributing effects to the intervention. I know that you mentioned control group but clearly state that is a major constraint to attributing effects to the intervention.

**Response:** We revised the limitations section to emphasize that the lack of a control group is a major constraint to attributing observed changes directly to the intervention on page 26, lines 572-573.

**New text in italics:** “*Most notably*, the study lacked a control group. *This major constraint limits our ability to attribute observed changes directly to the intervention, as we cannot rule out confounding.”*

**Writing and Clarity:**

Here, I will say that there are some aspects of the manuscript that contain grammatical errors or awkward phrasing, for example:

34. Page 9, Line 108: “so that there would not be any overlap”. Consider rephrasing to read “to avoid overlap in outreach areas.”

**Response:** In editing this section for clarity, this phrase has now been eliminated.

35. Page 27, Line 546: “...generally well-received”.  Please also consider saying “broadly acceptable and culturally appropriate.”

**Response:** In editing the Discussion for clarity, this sentence has now been eliminated.

36. I will suggest that a thorough copyedit is needed to correct minor grammar and enhance readability.

**Response:** We completed a thorough copyedit of the manuscript to improve readability and correct minor grammatical mistakes.

**Figures and Tables:**

37. For your Figure 1, I will suggest you ensure the figure is well-labelled and visually communicates the causal logic between intervention components and contraceptive agency outcomes.

**Response:** Figure 1 is the theory of action we developed as part of the human-centered design process that Ndingathe derived from; thus, we have replicated it here in the form we used for the pilot. In the text description of the Framework we provide more clarity about the two proposed mechanisms (see response to Comment # 18 above).

38. For your Tables 1 and 2 (Page 14), though they are information, I will still suggest you consider including a footnote indicating how missing data were handled (e.g., non-responses, incomplete forms). I have also included this also be addressed in methodology.

**Response:** Thank you for this suggestion. We included footnotes with each table detailing missing data (see Tables 1, 3, 4 pages 13, 15, 19).

**References:**

39. Again, please ensure that in-text references follow the PLOS ONE style, with reference numbers inserted *before* the full stop. Additionally, in the reference list, include the full DOI (<https://doi.org/>) or web address where applicable. I recommend consulting the journal's author submission guidelines for detailed instructions on formatting and referencing

**Response:** All references have been updated to follow the PLOS ONE style with references numbers inserted before the full stop.
